# Mental Health Symptoms Related to Body Shape Idealization in Female Fitness Physique Athletes

**DOI:** 10.3390/sports7110236

**Published:** 2019-11-14

**Authors:** Therese Fostervold Mathisen, Jorunn Sundgot-Borgen

**Affiliations:** 1 Faculty of Health and Welfare, Østfold University College, P.O. Box 700, 1757 Halden, Norway; 2 Department of Sport Medicine, Norwegian School of Sport Sciences, P.O. Box 4014, 0806 Oslo, Norway; jorunn.sundgot-borgen@nih.no

**Keywords:** aesthetic sport, disordered eating, body dissatisfaction, drive for leanness, drive for muscularity, exercise dependency, body ideal

## Abstract

Physical activity relates to optimal health, still the prevalence of mental health issues is high among athletes. Being young, female, and competing in aesthetic sports is a high-risk combination for mental health symptoms. Fitness physique athletes (FA) match this profile but are understudied. We aimed to study the intensity of mental health symptoms (i.e., body image, eating behaviour, relation to and routines for exercise, and perfectionism) in FA and in female references (FR), and to evaluate how preparing for fitness sport competitions affects these mental health symptoms. Before competition, FA had higher levels of drive for leanness (DFL) and eating restraint compared to FR. At the time of competition, eating restraint increased in FA only, concurrent with a reduction in symptoms of disordered eating. The levels of DFL, drive for muscularity, eating restraint, and exercising for figure toning were higher in FA compared to FR. At one-month post-competition, the differences between groups from competition time remained. Generally, perfectionism correlated with eating restrictions in FA and with disordered eating in FR. Overall, FA coped with the dieting, but self-control deteriorated post-competition with higher levels of disordered eating and an increased body shape concern. High DFL generally associated with more disordered eating behaviour, specifically in FR.

## 1. Introduction

Mental health symptoms and disorders are on rise in the young, Scandinavian population and are specifically prevalent in females, with a current point prevalence of self-reported mental health issues of 31% [1]. Physical activity associates with better mental health, specifically affecting body image, self-confidence and -efficacy, and symptoms of depression positively in adolescents and young adults [1,2,3,4,5]. Nevertheless, mental health impairments are also prevalent amongst athletes [6,7,8,9], a scenario endorsed by the International Olympic Committee consensus report on mental health symptoms and disorders in adolescent elite athletes [10].

A recent meta-analysis identified mental health symptoms and disorders as highly prevalent among elite athletes (34%) [6] and slightly higher compared to the frequency of mental health disorders in the general population (~20%) [11]. In line with findings in the general population, female athletes seem more prone to mental health issues than male athletes [12], and age seems to play a role, as adolescent athletes are reported to be more disposed when compared with adult athletes [8,12]. Furthermore, athletes competing in aesthetic sports seem to be more likely to report mental health symptoms or disorders compared to other athletes [8]. A reasonable explanation for the high prevalence in aesthetic sports may be an experience of high psychological pressure due to expectations of both sport performance and leanness and/or a specific body weight [13,14]. The latter associates to body weight and shape concerns [13,15], increasing the risk of unhealthy practices like excessive exercise volume and/or restrictive eating regimens [13,16,17]. Ultimately, such practices may result in relative energy deficiency [18]—a condition that affects physical health and performance, further increasing the risk of mental health symptoms and disorders [18].

Many athletes are high-achieving perfectionists, and there may be a reasonable hypothesis of a higher level of perfectionism in athletes, as this may be a favourable mental characteristic in order to make the necessary priorities and efforts to perform successfully [19]. This is great when it comes together to victory or a perfect performance, but the consequences of perfectionism can be tough when the results do not match an athlete’s own expectations [19]. High levels of perfectionism may bring up additional concerns for mental health in athletes, considering the association between perfectionism and the risk of eating disorder in the general population [20,21] and findings of correlation between perfectionism and exercise dependence in both athletes and those active for recreation [19]. Additionally, body image idealisation has generally been found to increase body dissatisfaction and to cause destructive exercise routines (e.g., obligatory exercise or exercise dependency) and disordered eating (DE) behaviour [22,23,24,25,26]. Hence, a strong idealisation of the “healthy athletic body ideal” (i.e., leanness and muscularity) may also be expected to be destructive to mental health. Still, there is some controversy on how these mental health aspects correlate in athletes and whether they are destructive or combined traits that strengthen the athletes, enabling them to perform optimally [9,16,19,20,26,27,28,29].

Fitness physique athletes compete in a range of fitness sport categories, all being subjectively evaluated in aesthetics, appearance, and performance on stage by a panel of nine judges [30,31]. The sport is increasing in popularity [31] and appeals specifically to young females by consolidating with the modern female, athletic body ideal [32,33]. This sport is a member of the International Federation of Bodybuilding and, as such, stands outside the organised international sport federations. This implies that athletes lack support from professional health teams, and, specifically, mental health issues are under-recognised challenges within this sport [31]. A few reports, mainly based on case studies or male participants, have revealed body dissatisfaction and DE behaviour as frequent issues among these athletes, with the post-competition period being especially challenging [31]. There is little knowledge on how such sport participation affects females, other than a few publications reporting transient physical health impairments during and after dieting for competition [31]. However, we recently identified a risk of undetected psychological issues in female fitness athletes, with 35% reporting a history of eating disorders and an increase in symptoms of mood impairment during the competition preparation period [34]. Based on the fact that age, gender, and this type of sport specifically relate to mental health symptoms, these young females, competing in purely aesthetic sports, might be recognised as a high-risk group.

Considering the growing popularity of fitness physique sports, and the fact that these athletes serve as the new, healthy body ideal for young women [31,35,36], we find it important to increase the knowledge on mental health symptoms in females who comply with such a lifestyle and at the same time participate in competitive fitness sports. As such, the aim of this paper was to explore the status of mental health symptoms (i.e., body image, eating behaviour, relation to and routines for exercise, and perfectionism) in young, female fitness athletes (FA), and to study how body image idealisation and eating and exercise behaviours correlate. Further, we aimed to study how a competitive period, which includes considerable energy restriction, may affect their body image, eating behaviour, relation to and routines for exercise, and level of perfectionism. In order to evaluate whether the findings are representative for the modern, young, physically active female or specific to this aesthetic sport, a reference group of recreationally active females (female references, FR) was included. We hypothesised the following: (1) the specific mental health symptoms are comparable between FA and FR at baseline; (2) during the dieting period for the FA (3–4 months), differences arise between FA and FR in mental health symptoms, with a deterioration in FA only; (3) shortly after the dieting period (i.e., one month post-competition), FA are still different from FR on outcomes related to body image and eating behaviour, but not in level of perfectionism or relation to and routines for exercise; and (4) high levels of body image idealisation correlate positively to destructive exercise routines (i.e., exercise dependence) and DE behaviour.

## 2. Materials and Methods

### 2.1. Design and Ethics

This is a cohort study conducted at the Norwegian School of Sport Sciences (NSSS) during 2017, measuring health variables in FA during a competition preparation period of 3–4 months and at one month post-competition. In addition, similar health variables were measured in a group of FR during the same time span. We performed no power calculation due to the explorative approach of this study.

All participants responded to questionnaires three times: at baseline (T1), before the energy-reduced diet was initiated; at two weeks pre-competition (T2), after following a three-month dieting period; and then finally at one month post-competition (T3). The three evaluation periods constituted a comprehensive test battery including both physical evaluation and standardised psychological questionnaires, but for the purpose of this paper, only results related to mental health symptoms or disorders are presented (see Mathisen et al. 2019 for physical health measures) [34].

The study was approved by the Norwegian Regional Committee for Medical and Health Research Ethics (ID: 2016/1718) and prospectively registered in Clinical Trials (ID: NCT03007459). All participants received written and oral information of the aim of this study and signed an informed consent before participation.

### 2.2. Participants

**Figure 1:**
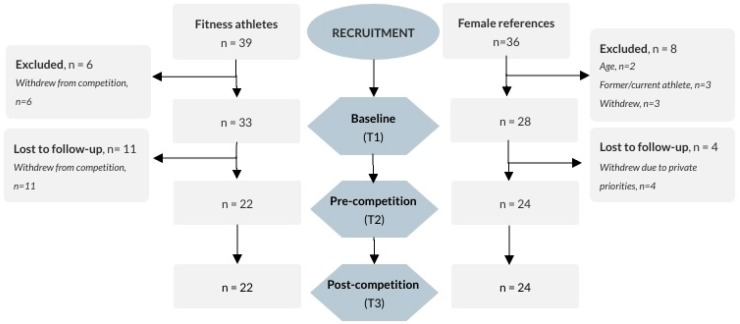
Overview of numbers of participants recruited, included and excluded, or lost to follow-up in the two groups at the three evaluation timepoints.

We recruited participants through a webpage distributed via social media, targeting both FA and FR, and by contacting all registered coaches officially listed within the Norwegian Federation of Fitness and Body Building. All responding FA planning to participate in the upcoming Norwegian competition period and initiating an energy-restricted diet for such attendance, aged 18–40 years, were included. FR at comparable age, with a body mass index (BMI) of 18.5–35, and with no former or planned future experience with fitness or bodybuilding sports, or current profession as a personal trainer, were included. An additional criterion for FR was being recreationally physically active within any kind of sport/activity at least two times per week during the current year. In total, 39 FA and 36 FR responded to recruitment, of which all who completed the questionnaires at baseline (33 and 28, respectively) were included in the analyses in this paper (see Figure 1).

### 2.3. Questionnaires

#### 2.3.1. Drive for Leanness (DL)

DL (Cronbach’s α = 0.87 in FA and 0.90 in FR) was developed as an alternative to the Eating Disorder Inventory (EDI) subscale “drive for thinness” to measure body dissatisfaction related to the lean athletic body ideal [37]. The scale reveals gender invariance, and there are findings of optimal validity [38]. It constitutes six questions scored by a 6-point scale ranging from never (1) to always (6) with a possible total score of 0–36, in which a higher score is indicative of greater investment in leanness. The total score in the current analysis is presented as the average item score (i.e., total score from 1 to 6).

#### 2.3.2. Drive for Muscularity (DM)

DM (Cronbach’s α = 0.88 in FA and 0.89 in FR) was created to measure any preoccupation with increasing muscularity and has revealed optimal reliability and construct validity [39,40]. It constitutes seven questions scored on a 6-point scale ranging from (1) always to (6) never, uses reversed coding in analysis, and has a total score of 15–90 (high score means higher DM). The total score in the current analysis is presented as the average item score (i.e., total score from 1 to 6).

#### 2.3.3. The Eating Disorder Examination Questionnaire (EDE-q)

The EDE-q (Cronbach’s α = 0.91 in FA and 0.96 in FR) comprises 22 items scored 0–6 to measure the presence and 6 items scored openly to measure the frequency of core ED characteristics [41]. It results in a global score (average item score; 0–6) and four subscale scores (body weight concern, body shape concern, eating concern, and eating restriction), in which a higher score means higher level of clinical severity. Global scores were presented in a previous publication [34]; hence, only subscales are presented herein. Additionally, the frequency of binge eating (EDE-q 14) and use of different purging methods (self-induced vomiting, use of laxatives, and driven exercise, i.e., EDE-q 16–18) are presented.

#### 2.3.4. The Binge Eating Scale (BES)

The BES (Cronbach’s α = 0.89 in both groups) was originally designed and validated to identify binge eaters within obese populations [42,43]. The BES constitutes 16 questions with ratings from 0 to 2 or 3, with total score 0–46 and higher scorings indicating higher clinical severity. A cut-off of ≥17 has been suggested to indicate the presence of any clinically meaningful binge-eating behaviour [42].

#### 2.3.5. The Three-Factor Eating Questionnaire (TFE-q 21)

The TFE-q-21 (Cronbach’s α = 0.90 in FA and 0.92 in FR) is an abbreviated version of the TFE-q-51 which was originally developed to measure symptoms of cognitive restrictive eating (RE, 6 items), emotional eating (EE, 6 items), and uncontrolled eating (UE, 9 items) in obese participants [44]. It constitutes 20 question rating agreements with different assertions by a score of 1–4, and one question rating agreement by 1–8 points, all being summarised in a total score and three subscale scores. RE measures the tendency to control food intake in order to influence body weight, EE measures the propensity to overeat in relation to negative mood states, and the UE measures the tendency to lose control over eating when feeling hungry or with external cues. The total subscale scores may be presented as raw scores or as transformed scores (scale 0–100); we used the latter method in the current study. A revised version, TFE-q-18, has also proved valid in a general population (e.g., all levels of body weight) [45].

#### 2.3.6. Reason for Exercise Inventory (REI)

REI (Cronbach’s α = 0.91 in FA and 0.83 in FR) was created to understand the reasons and motivation for exercise in female and male adults [46]. It consists of 26 items rated on a 7-point Likert scale ranging from 1 (not important) to 7 (extremely important) and is summarised in 7 subscales according to exercise motives using separate mean scores (subscales: weight control, fitness, mood, health, attractiveness, enjoyment, and tone).

#### 2.3.7. The Exercise Dependence Scale (EDS)

EDS (Cronbach’s α = 0.83 in FA and 0.93 in FR) is a validated instrument intended to measure dependency on exercise [47]. It constitutes 21 questions rated on a 6-point Likert scale (from 1—never to 6—always) and can be summarised as a global score and 7 subscale scores (only the global score is presented in the current study). The total score ranges from 21 to 126.

#### 2.3.8. The Child and Adolescent Perfectionism Scale (CAPS)

CAPS (Cronbach’s α = 0.91 in FA and 0.93 in FR) is a 22-item measure of perfectionism in children and adolescents, comprising two subscales: self-oriented perfectionism (12 items) and social-oriented perfectionism (10 items) [48,49]. It is an adapted version of the full-scale questionnaire for adults (Multidimensional Perfectionism Scale) [50], excluding the third scale on other-oriented perfectionism (not of interest to this study). Questions are rated from 1 (false—not at all true) to 5 (very true for me). CAPS has been evaluated as a reliable instrument for research purposes, and CAPS has been used for age groups up to at least 25 years of age [51,52].

### 2.4. Statistics

SPSS version 24 (IBM, Armunk, NY, USA) was used for statistical analyses. Demographic data from baseline were analysed by independent *t*-test, Mann–Whitney *U*-test, or chi-square analyses as appropriate, with a significance level of *p* < 0.05.

Within-group changes (T1 vs. the two other measures) and between-group differences (FA vs. FR) were analysed by linear mixed regression models. We included a random intercept factor to account for dependency in the repeated outcome measures, and standard errors were estimated with the restricted maximum likelihood function. Fixed factors in analyses were Group (FA, FR), Time (T1, T2, T3), and Group × Time. We examined differences between the groups with planned comparisons at each time point, and the within-group analyses included all three measurements in the factor Time. Differences with *p*-values of ≤0.01 were considered significant in all within- and between-group analyses. For the dichotomous outcome variables, we used a comparable statistical approach: a generalised linear model using a binominal distribution and logit link function. Degrees of freedom were computed using Satterthwaite approximation. All data are presented as estimated means with 99% confidence intervals. Correlations of variables to the global EDE-q score (i.e., symptoms of eating disorders) and to eating behaviour (BES and TFE-q 21) were analysed separately for each group by time with Pearson correlation.

We calculated standardised Hedges’ *g* effect sizes for continuous data as a ratio of the estimated means (extracted from the mixed model) to the observed pooled standard deviations (SD). Values of 0.2, 0.5, and 0.8 were interpreted as weak, medium, and strong effect sizes, respectively.

## 3. Results

A total of 33 FA and 28 FR responded to the baseline (T1) questionnaires (Figure 1). Demographic information on the participants is presented in Table 1. There were no differences in demographics or results from T1 between completers and drop-outs, in either FA (*p* > 0.1) or FR (*p* > 0.2). There was a significant difference between groups with respect to whether they received professional exercise guidance or were self-guided (*p* < 0.002). In FA, 18 (54%) were guided by a coach, 3 (9%) by a personal trainer, and 12 (36%) were operating their exercise routine individually. The corresponding numbers in FR were 3 (11%), 6 (21%), and 19 (68%).

### 3.1. Drive for Leanness (DFL)

**Figure 2:**
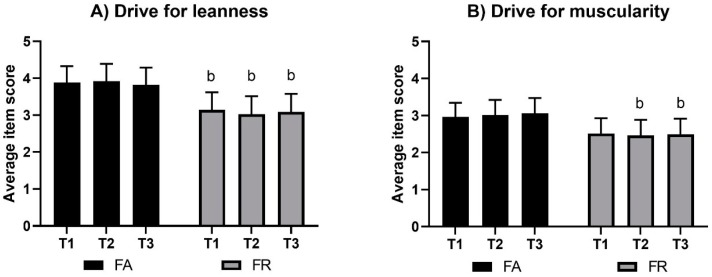
Scores in drive for leanness (**A**) and drive for muscularity (**B**). Values are average item scores (99% CI). Note: FA, fitness athletes; FR, female references; T1, baseline; T2, pre-competition; T3, post-competition; b, significant between-group difference, *p* < 0.01.

DFL results by group and by time are presented in Figure 2. There were no significant changes within groups by time; however, FA and FR were significantly different at all the three measurements (*g* = 0.8 at T1, 1.1 at T2, and 0.9 at T3; *p* < 0.005).

### 3.2. Drive for Muscularity (DM)

DM results by group and by time are presented in Figure 2. There were no significant changes within groups by time; however, lower mean (99% CI) scores in FR compared to FA were found at T2, with −0.6 ((−1.1, 0.0); *g* = 0.7, *p* = 0.01), and at T3, with −0.6 ((−1.2, 0.0); *g* = 0.7, *p* = 0.01).

### 3.3. The Eating Disorder Examination Questionnaire (EDE-q)

#### 3.3.1. Body Weight Concern (EDE-q)

The mean (99% CI) score for body weight concern at T1 was 1.0 (0.4, 1.6) in FA and 1.4 (0.7, 2.0) in FR, with no significant changes within groups (*p* > 0.02), nor any between-group differences at any time (*p* > 0.2).

#### 3.3.2. Body Shape Concern (EDE-q)

**Figure 3:**
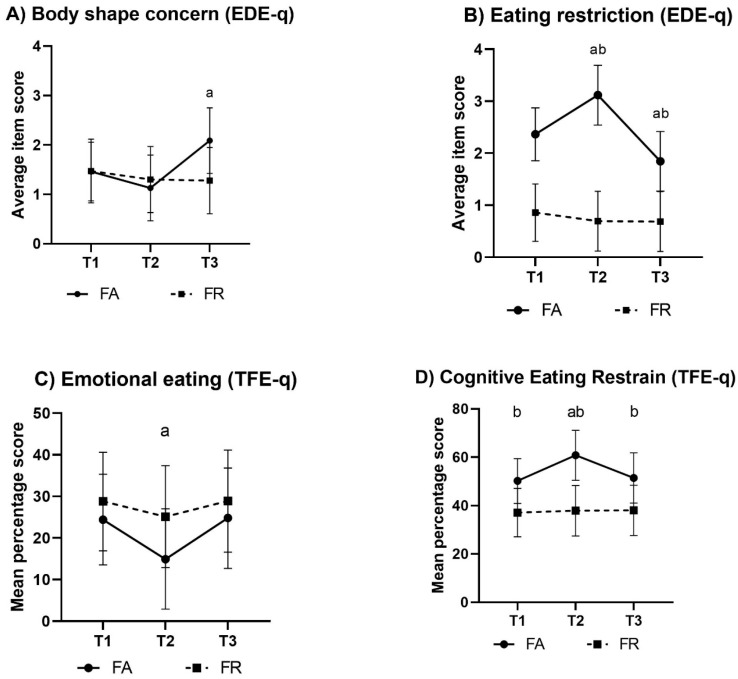
Changes in body shape concern and in eating restriction (both Eating Disorder Examination Questionnaire (EDE-q) subscales, illustrations (**A**,**B**), respectively) and in emotional eating and cognitive eating restraint (both Three-Factor Eating Questionnaire (TFE-q) subscales, illustrations (**C**,**D**), respectively) within groups. The values of the EDE-q subscales are mean subscale scores (99% CI), and those of the TFE-q subscales are mean subscale scores transformed to a 0–100% scale (99% CI). Note: FA, fitness athletes; FR, female references; T1, baseline; T2, pre-competition; T3, post-competition; a, significant within-group change in FA, *p* < 0.01; b, significant between-group difference, *p* < 0.001.

The results on body shape concern are presented in Figure 3. FA showed increased body shape concerns towards T3 by 0.6 (0.1, 1.2) points (*g* = 0.4, *p* = 0.004). There were no other significant changes within groups (*p* > 0.12), nor were there any differences between groups at any time (*p* > 0.3).

#### 3.3.3. Eating Restriction (EDE-q)

The results on eating restrictions are presented in Figure 3. Compared to T1, eating restriction was increased in FA at T2 (*g* = 0.8, *p* < 0.001), decreased at T3 (*g* = 1.1, *p* < 0.008), and different to FR at all times (*g* = 1.2, 2.3, 1.0 at T1–T3, respectively; *p* < 0.001).

#### 3.3.4. Eating Concern (EDE-q)

The mean (99% CI) scores for eating concern at T1 were 0.4 (0.0, 0.8) in FA and 0.5 (0.0, 0.9) in FR. In FA, there was a tendency of a significant increase by 0.3 (−0.03, 0.64) points in eating concern at T3 (*p* = 0.017), but there were no significant changes within groups (*p* > 0.2) and no between-group differences at any time (*p* > 0.2).

#### 3.3.5. Binge Eating Episodes (EDE-q, Item 14)

**Figure 4:**
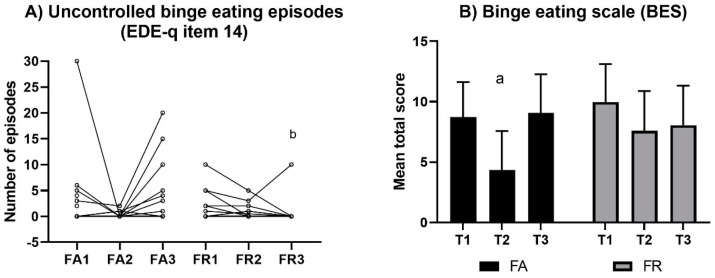
Individual variations in the reported number of uncontrolled binge eating episodes (**A**) and in mean symptoms of binge eating (**B**) within each group at each time point of evaluation. Values of Binge Eating Scale (BES) are the mean total score (99% CI). Note: FA, fitness athletes; FR, female references; T1, baseline; T2, pre-competition; T3, post-competition; FA1, baseline in FA; FA2, pre-competition in FA; FA3, post-competition in FA; FR1; baseline in FR; FR2, pre-competition in FR; FR3, post-competition in FR; a, significant within-group change (*p* < 0.001); b, significant between-group difference, *p* = 0.01.

The individual frequencies of binge eating episodes are presented in Figure 4. There was no significant change within groups in terms of binge eating episodes (*p* > 0.11); however, at T3 there was a significant difference between groups (*U* = 192, *p* = 0.01).

#### 3.3.6. Purging Episodes (EDE-q, Items 16–18)

There were few participants reporting the use of purging methods and, hence, no significant change by time or difference between groups. Four FA reported 2–10 episodes of self-induced vomiting across the study period, and two FR reported one episode each at T1. There was one FR reporting misuse of laxatives 10 times at T1 and one FA reporting four episodes at T1 and 12 episodes at T3. A total of 17 FR and 12 FA practiced driven exercise at T1 (episodes ranging from 1 to 50), while these numbers of participants were reduced to 6 and 8, respectively, at T2 (episodes ranging from 1 to 28) and to 7 FR and 7 FA at T3 (episodes ranging from 1 to 27).

### 3.4. Three-Factor Eating Questionnaire (TFE-q)

#### 3.4.1. Intensity of Uncontrolled Eating (TFE-q)

The mean (99% CI) percentage scores for uncontrolled eating at T1 were 31.8 (22.6, 41.1) in FA and 38.6 (28.6, 48.6) in FR, with no change within groups (*p* > 0.14) or any differences between groups at any time (*p* > 0.2).

#### 3.4.2. Intensity of Cognitive Eating Restraint (TFE-q)

The intensity of cognitive eating restraint is presented in Figure 3. In FA, the cognitive eating restraint increased towards T2, with a mean (99% CI) percentage score of 60.8 ((50.4, 71.1); *g* = 0.7, *p* = 0.003). There were no other within-group changes (*p* > 0.7); however, between-group differences existed for all time points (*g* = 0.7, 1.1, 0.6, respectively; *p* < 0.01).

#### 3.4.3. Intensity of Emotional Eating (TFE-q)

The intensity of emotional eating is presented in Figure 3. There was a significant reduction in emotional eating in FA at T2, with a mean (99% CI) of 14.9 ((2.9, 27.0); *g* = 0.8, *p* = 0.01). There were no other within-group changes (*p* > 0.30) or between-group differences (*p* > 0.12) at any time.

### 3.5. Symptoms of Binge Eating (BES)

The mean score in symptoms of binge eating decreased in FA from T1 to T2 (*g* = 1.2, *p* < 0.001), but no other significant changes were identified, nor were there any between-group differences at any time (*p* > 0.07) (Figure 4). The mean (99% CI) proportion above the BES cut-off at T1 was 9% (2, 31) in FA and 21% (8, 46) in FR, with no significant changes within groups (*p* > 0.06) or differences between groups at any time (*p* > 0.02).

### 3.6. Reasons for Exercise (REI)

The mean scores for each of the reason-for-exercise scales are presented in Table 2. Both groups overall rated health enhancement, mood regulation, and fitness function as important arguments for motivation to exercise, and only exercise for figure toning was different between the groups.

### 3.7. Exercise Dependency (ExD)

At T1, the mean (99% CI) scores in symptoms of ExD were 57.5 (51.2, 63.8) in FA and 54.0 (47.2, 60.8) in FR. There were no significant changes within groups by time (*p* > 0.21), nor were there any between-group differences at any time (*p* > 0.18).

### 3.8. Symptoms of Perfectionism (CAPS)

The mean (99% CI) scores for self-oriented perfectionism (CAPS-self) at T1 were 2.8 (2.5, 3.2) in FA and 2.8 (2.4, 3.2) in FR. The corresponding results for social-prescribed perfectionism (CAPS-social) were 2.2 (1.9, 2.6) and 2.2 (1.9, 2.6), respectively. There were no significant changes in scores of CAPS-self or CAPS-social within either of the groups (*p* > 0.11), nor were there any between-group differences at any time (*p* > 0.18).

### 3.9. Correlates

Correlations (identified and presented as Pearson’s r) between eating behaviours and body image idealisation are presented in Table 3. Additionally, correlations between body image idealisation, ExD, and perfectionism were identified. DM was negatively correlated to ExD at all measurement points (−0.6, −0.7, −0.7; *p* ≤ 0.001) in FA and in FR (−0.7, −0.8, −0.8; *p* < 0.001). In FA, DM also correlated negatively to CAPS-self at all measurement times (−0.6, −0.5, −0.6; *p* < 0.1) and to CAPS-social at T1 and T3 (−0.5 and −0.6; *p* < 0.009), but no correlation was found between DM and perfectionism in FR. Concurrently, DFL correlated positively to ExD in FA at T1 and T2 (0.4 and 0.6, respectively; *p* < 0.1), and at all measurement points in FR (0.6, *p* < 0.002). In FA, DFL also correlated positively to CAPS-self at T1 and T3 (0.5 and 0.6, respectively; *p* < 0.006) and CAPS-social at T3 (0.5, *p* = 0.01), while only CAPS-self at T3 correlated in FR (0.5, *p* = 0.01). Finally, ExD correlated positively with CAPS-self in both groups (between 0.5 and 0.7, *p* < 0.008) and to CAPS-social in FA at T1 and T3 (0.5 and 0.6, respectively; *p* < 0.005). ExD also correlated positively with EDE-q in FA at T2 (0.5, *p* = 0.01) and at all measurement times in FR (0.6, 0.7, 0.7, respectively; *p* ≤ 0.001).

## 4. Discussion

We aimed to explore mental health symptoms in female fitness athletes (FA) and to study whether aspects of mental health were changed by extreme dieting and body composition changes leading up to a competitive season. A female reference group (FR) was included to control for the effect of body image idealisation in modern society and for the effect of time. In contrast to our first hypothesis suggesting similarities in all mental health measures between groups at baseline, we found higher scorings in FA in drive for leanness (DFL) and in cognitive eating restraint, both with strong effect sizes. In accordance to the second hypothesis, at T2, eating restriction and cognitive eating restraint increased, and emotional eating and symptoms of binge eating decreased in FA only, all with strong effect sizes. Still, only eating restriction and cognitive eating restraint were significantly different between groups, additional to the higher drive for muscularity (DM), DFL, and exercising for figure toning in FA compared to FR (these differences also with strong effect size). Contrasting our third hypothesis, FA expressed increased body shape concern at T3 (medium effect size), still with no between-group difference. Nevertheless, there was a stronger motivation to exercise for figure toning in FA compared to FR. In line with the third hypothesis were the higher levels of eating restriction, cognitive eating restraint, DFL, and DFM in FA compared to FR at T3 (medium to strong effect sizes). Finally, in accordance to our last hypothesis, high levels of DFL correlated positively with exercise dependence (ExD) and symptoms of eating disorders (EDE-q) in both groups. DFL also correlated with uncontrolled and emotional eating in FR and with cognitive restraint in FA. Contrasting to the last hypothesis, was the finding of negative correlation between DM and ExD, and between DM and EDE-q,

Perfectionism usually correlates to ED [20,21] and might be a relevant issue in athletes representing sports in which EDs are common [16,17,20,53]. In the current study, perfectionistic strivings (i.e., self-oriented perfectionism) were somewhat higher than those reported in normative results, with the opposite observed for perfectionistic concern (i.e., socially prescribed perfectionism) [54]. Still, with a reported increase in perfectionism in the last decades [52], our results indicate moderate levels of perfectionism compared to more recent findings [52,55,56]. Interestingly, most correlates of perfectionistic outcomes were found in FA, and very few were found for FR. This may implicate a stronger role of perfectionism in motivation for sport performance in these athletes, which is in line with previous findings of perfectionistic tendencies in athletes [15,19]. The literature suggest that perfectionism concerns bring more clinical concerns, while perfectionism strivings might serve as a positive trait [15,52,55]. We found correlations between self-oriented perfectionism, leanness idealisation, and both eating restriction and cognitive restriction in FA, but also between socially oriented perfectionism and eating and cognitive restrictions. This may imply that FA have high perfectionistic strivings in order to comply with the lean athletic body ideal but do also experience pressure of expectations from their social network. In FR, the correlations of perfectionism scales were exclusively to less healthy eating behaviour, i.e., emotional and uncontrolled eating, and between social perfectionism and DFL. This may implicate external motivations, with strivings towards a socially accepted body ideal and with experiences of failure, resulting in increased uncontrolled eating behaviour.

Since the introduction of the DM questionnaire two decades ago, several publications have revealed a steady female idealisation of the athletic body over time [57]. Our results are comparable to the strength of symptoms in these previous publications; however, FA favour such a body ideal more strongly than females not adhering to fitness sports (FR). The same scenario relates to the DFL, in which our FR sample was comparable to previous reports from large samples [58,59] but with a specifically strong idealisation of the lean, toned body ideal amongst FA. Recently, DFL was suggested to be less maladaptive compared to DM or drive for thinness in regards to unhealthy eating behaviour [60]. This contrasts to the present findings in which there is a positive association between symptoms of eating disorders and DFL and a concurrent negative association with DFM. Additionally, while the DFL correlates positively to ExD and DE behaviours in both FA and FR, the DFM correlates negatively. Hence, in line with previous publications, we found a less harmful impact from athletic, muscular idealisation compared to the idealisation of a thinner or lean body [61,62,63].

There seem to be important differences in how FA and FR deal with body image idealisation. In FA, a high score in DFL correlates with symptoms of eating disorders (EDE-q) and perfectionism. However, the relationship to eating behaviour indicates strong control during the dieting period (T1, T2), with positive correlations to eating restrictions and ExD and random or no correlation to DE (BES, TFE-q, and reported binge eating episodes). This implies an ability to follow the necessary restrictive procedures when motivation is high in order to succeed with contest preparations (i.e., to become lean and defined). Hence, extreme body image idealisation might serve as a strong motivator for extreme dieting and exercise regimens when there is an end to the efforts within proximity (i.e., in FA).

On the contrary, the FR seem to deal with the DFL differently, as correlation with symptoms of eating disorders (EDE-q) accompanies correlations with symptoms of binge eating (BES), uncontrolled eating episodes, and emotional eating episodes (both TFE-q). Additionally, in FR, DFL correlated to ExD at all times, with the latter also correlated to symptoms of eating disorders (EDE-q) at all times. Hence, there seems to be a risk of unhealthy coping strategies when there is a chronic exposure to body idealisation concurrent with an experienced distance to the ideal (i.e., in FR).

Of importance for the overall interpretation of how fitness sport participation might affect mental health are the post-competition findings of mental health symptoms in FA. A previous publication from the current sample identified a temporary decrease in energy intake between T1 and T2, which returned to baseline at one month post-competition [34]. This post-competition increase in energy intake was accompanied by a quick restoration of body weight and fat mass [34]. As such, despite the current findings of continued higher cognitive eating restraint and eating restriction in FA compared to FR post-competition, the FA were not able to avoid the increase in energy intake and body weight [34]. At the same time, FA increased their body weight concern, eating concern, driven exercise, and exercise motivated by figure toning—all coinciding with increased symptoms of binge eating (BES) and actual number of binge eating episodes. Adding to the concern of these cognitive and behavioural changes in FA are the findings of a high frequency of self-induced vomiting and the use of laxatives in some of the athletes. The latter (i.e., purging behaviour) was also previously reported as a more frequent issue in elite athletes compared to controls [53] and may be a result when athletes experience a loss of control of food intake or reduction in a long period of eating restrictions, also increasing their worries for body weight and shape.

Overall, our findings suggest adaptive coping strategies in competitive FA, concurring with findings of favourable mental health status in young athletes compared to controls [9]. However, the strict dieting and exercise regimens cannot be maintained for a long time, and as such, body shape deteriorates from the idealised body image. Previously, such deteriorations were found to increase maladaptive eating and exercise behaviour in the long term in athletes [64]. Based on our findings on the strong, immediate mental health deteriorations post-competition, and considering the potential long-term effects, there are reasons for concern when it comes to mental health in FA. The current findings, with increased DE post-competition, do also imply a warning against participation in fitness sport competitions if having a history of (or current) eating disorder.

The limitations to our findings are the use of questionnaires developed and primarily validated for other population samples (i.e., BES and TFE-q for obese persons; CAPS for adolescents; DFL and DM for males), no clinical interviews, and a short follow-up period. Furthermore, we have reasons to believe that the FR had personal motives and interests in the topic of this trial, as the prevalence of self-reported history of eating disorders in this small sample was comparable to the lifetime prevalence in the general population [65]. As such, a biased reference sample may interfere with the interpretation of the findings in FA.

## 5. Conclusions

In summary, our findings suggest that FA are strongly inspired by the lean, athletic ideal (i.e., high DFL and DM) and are hence attracted to a competitive sport with high emphasis on such characteristics. The combination of high perfectionism and internalising of the lean, athletic body ideal results in high body weight and shape concerns. This motivates cognitive restrictions and exercising for figure toning and results in uncontrolled eating episodes. Additionally, those *not* internalising the lean, athletic body ideal to such an extent as those wanting to compete in fitness sport (i.e., FR) are still influenced by the societal overvaluation of such ideals. We found correlations between self-oriented perfectionism and body image idealisation in FA, but not in FR. This finding may be a potential moderator of how DFL differentially correlates to eating behaviour, with more cognitive and eating restrictions in FA and more uncontrolled and emotional eating in FR. Interpretation of the clinical severity of our findings is limited by the lack of long-term follow-up, and as such, longstanding studies specifically on mental health symptoms in FA are warranted.

## Figures and Tables

**Table 1:** Demographic information on participants at baseline (T1). Values are presented as the mean (Sd), and effect sizes on any differences are presented with Hedges’ *g*.

Demographics	Female Athletes, *n* = 33	Female References, *n* = 28	p-Level, Effect Size (g)
Age, years	28.4 (5.6)	30.2 (6.0)	0.23
BMI, kg × m^−2^	22.6 (2.0)	23.1 (2.8)	0.42
Bodyfat, %	24.6 (5.9)	28.4 (5.7)	0.02, *g* = 0.65
Previous eating disorder *, *n* (%)	9 (28%)	3 (11%)	0.09
Current eating disorder *, *n* (%)	2 (6%)	1 (4%)	0.89
EDE-q, total score	1.3 (0.8)	1.0 (1.1)	0.30

NOTE: * information on previous and current eating disorders is self-reported. The discrepancy in frequency of ED between current paper and previous publication [34] is due to different number of participants included in baseline measures.

**Table 2:** Subscale scores in the reason for exercise inventory (REI) in fitness athletes and female references. Scores range from 1 to 7, with higher scoring indicating higher valuation. Values are average item scores (99% CI), and the effect size is Hedges’ *g*.

REI Subscale	Fitness Athletes			Female References		
	T1 *n* = 33	T2 *n* = 22	T3 *n* = 22	T1 *n* = 28	T2 *n* = 24	T3 *n* = 24
Weight control	2.3 (1.8, 2.8)	ns	ns	2.4 (1.8, 2.9)	ns	ns
Fitness function	4.5 (4.1, 5.0)	ns	ns	4.4 (3.9, 4.9)	ns	ns
Mood regulation	4.8 (4.2, 5.4) -	4.2 (3.5, 4.9) *p* = 0.005 *g* = 0.5	ns	5.0 (4.3, 5.6) -	4.2 (3.5, 4.9) *p* = 0.001 *g* = 0.8	4.3 (3.7, 5.0) *p* = 0.002 *g* = −0.1
Health enhancement	4.8 (4.3, 5.3)	ns	ns	4.8 (4.3, 5.3)	ns -	ns -
Attractiveness	3.4 (2.8, 4.0) -	2.9 (2.2, 3.5) *p* = 0.008 *g* = 0.5	ns	3.0 (2.4, 3.6)	ns	ns
Enjoyment	4.1 (3.6, 4.6) -	ns	ns	3.8 (3.2, 4.4) -	4.3 (3.7, 4.9) *p* = 0.01 *g* = −0.6	ns
Figure toning	3.7 (3.1, 4.2) ^$^ -	3.8 (3.2, 4.5) * ns	4.0 (3.3, 4.6) ^#^ ns	2.8 (2.2, 3.5) ^$^ -	2.9 (2.3, 3.6) * ns	2.9 (2.2, 3.5) ^#^ ns

NOTE: T1, baseline; T2, pre-competition; T3, post-competition; ns, non-significant within-group change; *g*, Hedges’ *g*; ^$^ significant between-group difference, *g* = 0.8, *p* = 0.01; * significant between-group difference, *g* = 0.6, *p* = 0.01; ^#^ significant between-group difference, *g* = 0.9, *p* = 0.002.

**Table 3:** Correlations to disordered eating behaviours and to symptoms of eating disorders (EDE-q) at the three different assessment time points (T1–T3), separated by groups (FA vs. FR). Only values for variables identified as significant correlates (*p* < 0.01) are presented (not presented, or empty cells, means no significant correlation). Values are Pearson’s r (above) and *p*-values (below).

Correlations		EDE-q			BES			UE			EE			CR			EC			ER		
		T1	T2	T3	T1	T2	T3	T1	T2	T3	T1	T2	T3	T1	T2	T3	T1	T2	T3	T1	T2	T3
DFL	FA	0.4 0.01	0.6 0.002	0.6 0.003					0.5 0.01					0.5 0.002	0.6 0.008	0.7 0.001		0.5 0.01		0.4 0.01		
	FR	0.5 0.007	0.7 <0.001	0.6 0.002		0.6 0.002	0.6 0.002		0.6 0.001	0.5 0.01		0.6 0.005	0.6 0.002				0.5 0.003	0.6 0.002	0.5 0.006		0.5 0.01	
DM	FA	−0.5 0.009		−0.6 0.007				−0.5 0.004						−0.6 0.001	−0.5 0.01	−0.6 0.001						
	FR	−0.5 0.005	−0.7 <0.001	−0.7 <0.001					−0.8 <0.001			−0.6 0.007	−0.7 0.001		−0.5 0.01		−0.6 0.001	−0.8 <0.001	−0.7 <0.001		−0.6 0.003	−0.6 0.002
C-self	FA	0.5 0.004			.5 .006									0.6 <0.001	0.5 0.009							
	FR									0.5 0.007												
C-social	FA			0.6 0.002										0.5 0.003	0.6 0.006						0.6 0.004	0.6 0.004
	FR									0.5 0.008			0.5 0.01									

NOTE: FA, fitness athletes; FR, female references; EDE-q, Eating Disorder Examination Questionnaire; BES, Binge Eating Scale; UE, uncontrolled eating (TFE-q); EE, emotional eating (TFE-q); CR, cognitive restraint (TFE-q); EC, eating concern (EDE-q); ER, eating restriction (EDE-q); ExD, exercise dependency; DM, drive for muscularity; DFL, drive for leanness; C-self, CAPS self-oriented; C-social, CAPS socially oriented; TFE-q, Three-Factor Eating Questionnaire; CAPS, Child and Adolescent Perfectionism Scale; T1, pre-competition; T2, at competition time; T3, post-competition.
